# Real-Time Continuous Glucose Monitoring Reduced Costly Diabetes-Related Events in Adolescents and Young Adults despite Lack of Short-Term Reduction in Hemoglobin A1c

**DOI:** 10.1155/2023/5253515

**Published:** 2023-04-11

**Authors:** Joni K. Beck, Rebecca A. Allen, Kathryn M. Jeter, Rachel S. Fisher, Taylor M. Dattilo, Katherine A. Traino, Michael Anderson, James Cutler, David P. Sparling

**Affiliations:** ^1^Section of Pediatric Diabetes & Endocrinology, University of Oklahoma Health Sciences Center and the OU Health Harold Hamm Diabetes Center, Oklahoma City, OK, USA; ^2^Section of General & Community Pediatrics, University of Oklahoma Health Sciences Center and the OU Health Harold Hamm Diabetes Center, Oklahoma City, OK, USA; ^3^Department of Psychology, Oklahoma State University, Stillwater, OK, USA; ^4^Department of Biostatistics and Epidemiology, College of Public Health, University of Oklahoma Health Sciences Center, Oklahoma City, OK, USA

## Abstract

Real-time continuous glucose monitoring (rtCGM) can directly improve patient outcomes, including decreased health care system utilization and associated costs. The purpose of this study was to evaluate the clinical benefits of rtCGM use in a high-risk, under-resourced cohort of adolescents and young adults (AYA) with type 1 diabetes (T1D) who had no prior access to rtCGM. The effects of rtCGM use on hemoglobin A1c (A1c) and the frequency of health care events (i.e., diabetes-related emergency room (ER) visits, hospitalizations, emergency medical services (EMS), and after-hour emergency calls) were evaluated regarding payor costs in 33 AYA with ≥70% rtCGM use. Secondary aims included the evaluation of a phone-based pattern management intervention. The frequency of health care events decreased at 12 and 24 weeks for all participants, and there was no significant difference by treatment group. We estimated that the use of rtCGM in this cohort results in a projected annualized cost-savings of $195,943 to $294,864 or 43–65% per year based on Medicare or list pricing for rtCGM, respectively. Results also revealed improvements in A1c at 12 weeks for all study participants, but this was not maintained at 24 weeks for the phone-based pattern management intervention group. Our findings suggest that rtCGM may be an effective tool for reducing diabetes-related events and underscores the importance of access. Future studies are needed to further examine tailored interventions and support to optimize rtCGM use and glycemic health in high-risk AYA.

## 1. Introduction

Technological innovations provide an opportunity to decrease the self-management burden and improve outcomes for individuals with type 1 diabetes (T1D). Research shows that individuals using continuous glucose monitoring (CGM) show lower hemoglobin A1c (A1c) values and increased time spent in range, regardless of the treatment regimen [1–7]. Adolescents and young adults (AYA) with T1D are tasked with increased self-management during an already challenging transition period navigating educational, vocational, and relational changes [8]. During this transition, AYA are at an increased risk for health-related difficulties [9]. The transition from pediatric to adult care has been identified by the American Diabetes Association (ADA) as a high-risk period of life for individuals with T1D [10] due to worsening glycemic health and an increased risk for acute complications, including hypoglycemia and diabetic ketoacidosis (DKA) [11]. The ADA first recommended CGM use for all children and adolescents with T1D in 2018 [12] and continues to endorse this recommendation [13]. However, despite CGM now being accepted in global standards of care [14, 15], it is still underutilized in AYA for a multitude of reasons, including device intricacies, cost-prohibitive access, or various social determinants of health and related disparities [16–20].

Research has shown a relationship between CGM and a decrease in hospitalizations [21]. Specifically, CGM has been shown to lower inpatient admissions of diabetic ketoacidosis (DKA) in adults with T1D [22]. Additional research has found that individuals with continuous subcutaneous insulin infusion (CSII) and CGM experience significant reductions in the occurrence of diabetes-related hospitalizations compared to individuals with CSII and the use of traditional blood glucose monitoring [23]. Diabetes care costs continue to rise irrespective of advanced treatment options, as is illustrated by DKA admissions in the United States, now over $26,000 per admission [24]. In addition, costly diabetes-related health care expenditures have been related to a multitude of social determinants of health that warrant targeted interventions to decrease health disparities [25, 26]. CGM has been discussed as a conceivable tool to decrease DKA and other diabetes-related events, but further evaluation is needed to understand the effectiveness of CGM use and acute health care costs in a high-risk AYA population.

The purpose of this study was to evaluate the health and cost benefits of CGM use in a high-risk, resource-challenged cohort of AYA with T1D who had no prior access to real-time continuous glucose monitoring (rtCGM). In this analysis, we evaluate the effects of rtCGM use on A1c and diabetes-related events (i.e., diabetes-related emergency room (ER) visits, hospitalizations, emergency medical services (EMS), and after-hours emergency calls), specifically looking at cost impact. A secondary analysis was to determine if a phone-based pattern management intervention might impact the results.

## 2. Materials and Methods

### 2.1. Participants

Participants were considered a high-risk group for AYA in that they had limited financial and social resources that prevented access to rtCGM. Limited access was due to no insurance or insurance policies where rtCGM was either cost-prohibitive or inaccessible due to unmet conditions (e.g., not checking fingerstick glucose values enough each day). All AYA meeting inclusion criteria were recruited from an endocrinology clinic in consecutive order between May 2020 and March 2021. Study participants had previously completed diabetes education, recognized by the ADA for its pediatric diabetes self-management education and support (DSMES) services [27]. In addition, all AYA received ongoing education updates, as needed, at their regular outpatient clinic visits and had 24/7 access to the pediatric team, as is typical of most pediatric diabetes centers.

Inclusion criteria included those 15–25 years of age with a clinical diagnosis of T1D ≥1 year, naïve to rtCGM, the ability to speak and read English, and at least one diabetes-related event (defined as any diabetes-related ER visit, hospitalization, use of EMS services, or after-hour emergency call in the past 18 months). Events at baseline were gathered and confirmed by two study team members from the patients' medical records or received from an outside healthcare facility. Exclusion criteria included intellectual disability, psychiatric hospitalization within the past 12 months, minors without a permanent guardian, and those unable to upload rtCGM data. Additionally, participants were excluded if they were taking glucocorticoids, had a history of hemoglobinopathies, and were pregnant or expected to become pregnant during the study duration.

### 2.2. Study Procedures

The protocol was approved by the institutional review board at the University of Oklahoma Health Sciences Center. Written informed consent from the participants, or parental consent and assent from patients who were 15 to <18 years of age was obtained. Participants were reconsented if they turned 18. A total of 52 participants were recruited for the study and were randomized into a treatment-as-usual (TAU) group or the phone-based pattern management intervention group. Participants in the TAU group received standard of care over the 24-week study period.

Participants in the intervention group, in addition to the standard of care, completed three phone-based pattern management education phone calls at 2, 4, and 8 weeks (set prior to COVID-related telehealth modifications). Phone calls were conducted by CDCES® study personnel who focused on the interpretation of CGM data and pattern management strategies. The phone calls were scheduled to spend interactive time discussing the CGM report with the participant and parent/legal guardian using standardized recommendations for interpretation. The intent was to see if additional pattern management discussions, realistic to conduct in a busy pediatric/young adult diabetes center, might help this high-risk AYA group have an improved understanding and application of their rtCGM data to further improve outcomes [27]. Each call lasted an average of 20–30 minutes.

All participants were provided Dexcom G6 sensors, transmitters, and a receiver (Dexcom, Inc.) at study onset and as needed throughout. The participants were allowed to use a personal mobile device, if preferred. They were were trained on the CGM device by certified trainers who followed standardized training procedures. Individuals on CSII therapy did not use the rtCGM with predictive low-glucose suspend or hybrid closed loop pump systems during the study. All study participants were instructed to call for any questions or concerns regarding the device or glucose trends.

The data was collected at baseline, 12 and 24 weeks. Study visits occurred on the same day as routine medical visits or within two weeks. Each medical visit consisted of a history, physical, and laboratory, as recommended by the ADA.

### 2.3. Clinical Measurements

All medical and demographic data were gathered from our healthcare systems electronic medical record (EMR) and compared at baseline, 12 and 24 weeks. A1c values (analyzed using a Bayer DCA 2000® Analyzer (Bayer Diagnostics Inc., Tarrytown, NY, USA; normal range 4.3–6.2%)), were also gathered at baseline, 12 and 24 weeks ± 2 weeks. Event data, both prior to intervention and post-rtCGM initiation, were collected at each clinic visit during provider assessment. In addition, study personnel verbally inquired and reviewed the healthcare systems' EMR, which encompassed adult and pediatric hospitals and clinics. If an event occurred at an outside health care facility, those medical records were obtained to confirm and appropriately classify the event.

### 2.4. Data Analysis

The data were analyzed to determine if a treatment effect would occur under recommended rtCGM conditions (i.e., device wear ≥70%). The data were excluded for a time point if the participant was unable to complete the scheduled study visit within ±2 weeks. Fifty-two participants were consented into the study. After 24 weeks, collectively, 9 participants either moved or were lost to follow-up. Five participants withdrew from the study for no longer wanting to wear a CGM device. One participant was removed from the study due to pregnancy. An additional 5 participants completed the study but were excluded from analyses due to insufficient device use. Thus, a total of 37 participants completed the study. However, of those, 33 participants completed the study with ≥70% rtCGM use and this data was used in the current analyses. For the secondary analysis, 29 participants were initially randomized to the intervention group and 23 in the TAU group. After accounting for dropout and sufficient rtCGM use, 20 participants in the intervention group and 13 participants in the TAU group completed the study, and this data were used in subsequent analyses.

### 2.5. Statistical Analysis

Descriptive statistics were computed for all demographic and clinical variables. Descriptive statistics for the overall sample, TAU and intervention group are summarized in Tables 1–4. Categorical variables were tabulated and summarized as frequencies (%), while continuous variables were assessed for normality using the Shapiro–Wilk test and summarized as Mean (±Standard Deviation) or Median (25th%, 75th%), as appropriate. Repeated measures analysis using linear mixed effects regression models were conducted to test for the independent effect of treatment group over time. The linear mixed model included a fixed effect for group and time and a random intercept for subject. Estimated model slope coefficients along with 95% confidence intervals were obtained. Student's *t*-test with appropriate degrees of freedom was used to test for significant slope coefficients and resulting *p* values were reported. The intraclass correlation coefficient (ICC) for each model run is computed to determine whether including the random subject effect improved the model fit for the observed data. Type III sums of squares are used for all hypothesis tests along with two-sided *p* values and significance level of 0.05. R version 4.2.1 with the lme4 package was used for the analysis.

## 3. Results

Analyses reflected that the groups were similar at baseline assessment in terms of demographics (e.g., race, age), A1c, insulin management, and diabetes-related events (*p* values = 0.086 to >0.900) (Table 1). Analysis performed examined the group as a whole against baseline, as well as between the intervention groups, across A1c, ER visits, hospitalizations, EMS use, and after-hours emergency calls (Table 2). At baseline, prior to rtCGM initiation, primary reasons for ER visits were hyperglycemia (56%), hypoglycemia (22%), 11% illness and hyperglycemia, and 11% out of insulin. Reasons for hospitalizations were DKA (62%), hyperglycemia management (31%), and 7% illness and hyperglycemia. The use of rtCGM at 12- and 24-week follow-ups were similar for the TAU and intervention group (*p* values = 0.508). CGM metrics, including mean glucose, time in range (TIR), and periods of hypoglycemia and hyperglycemia did not vary between the TAU and intervention groups at 12 weeks (Table 3) or 24 weeks (Table 4).

### 3.1. Mixed Effects Regression Models

#### 3.1.1. Diabetes-Related Events

**Figure 1:**
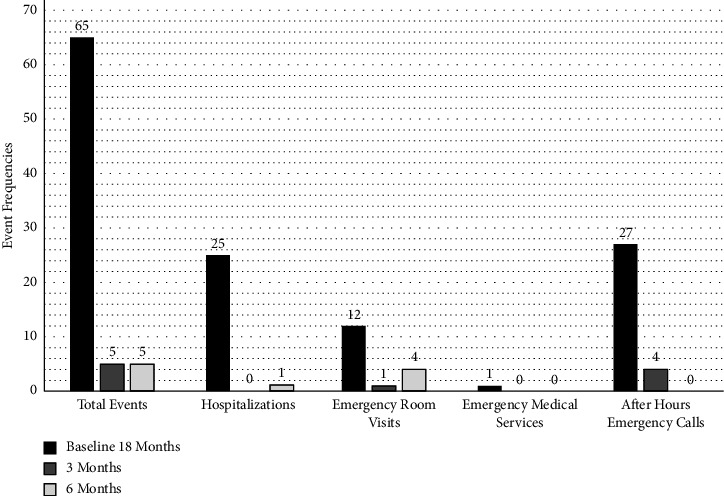
Events.

In the unconditional model, examining the whole cohort against baseline, the total frequency of diabetes-related events decreased significantly across the study for all participants at the 12-week follow-up (*b* = −1.76, *SE* = 0.25, *p*  <  0.001) and at the 24-week follow-up (*b* = −1.76, *SE* = 0.25, *p*  <  0.001). (Figure 1) In the final multivariate model, diabetes-related events significantly decreased by the 12-week (*b* = −1.85, SE = 0.39, *p* = <0.001) and 24-week (*b* = −1.62, SE = 0.39, *p* = <0.001) time points. In examination of our secondary aim, regression coefficients indicated that there was not a significant treatment group difference in diabetes-related events at baseline (*p* = 0.951). There were also not significant treatment group differences in the change in diabetes-related events from baseline to the 12-week (*p* = 0.928) or 24-week time points (*p* = 0.644) (Table 5). We then examined each diabetes event independently.

#### 3.1.2. ER Visits

Results from the unconditional model demonstrated that the occurrence of ER visits decreased at the 12-week follow-up (*b* = −0.33, *SE* = 0.11, *p* = 0.004) and the 24-week follow-up (*b* = −0.24, *SE* = 0.11, *p* = 0.034) across all study participants. In the final multivariate model, ER visits significantly decreased by the 12-week (*b* = −0.54, SE = 0.18, *p* = 0.003) time point but not the 24-week (*b* = −0.31, SE = 0.18, *p* = 0.088) time point. Regression coefficients indicated that there was not a significant treatment group difference in ER visits at baseline (*p* = 0.078). There were also not significant treatment group differences in the change in ER visits from baseline to the 12-week (*p* = 0.143) or 24-week time points (*p* = 0.639) (Table 5).

#### 3.1.3. Hospitalizations

In the unconditional model, hospitalizations decreased significantly over time for all participants at 12-week follow-up (*b* = −0.76, *SE* = 0.18, *p*  < 0.001) and 24-week follow-up (*b* = −0.73, *SE* = 0.18, *p*  < 0.001). In the final multivariate model, hospitalizations significantly decreased by the 12-week (*b* = −0.77, SE = 0.29, *p* = 0.009) and the 24-week (*b* = −0.69, SE = 0.29, *p* = 0.019) time points. Regression coefficients indicated that there was not a significant treatment group difference in ER visits at baseline (*p* = 0.944). There were also not significant treatment group differences in the change in ER visits from baseline to 12-week (*p* = 0.959) or 24-week time points (*p* = 0.877) (Table 5).

#### 3.1.4. EMS

Events for EMS were not analyzed separately due to small numbers. There was only one use of EMS for hypoglycemia at baseline and none in the study. The cost of this event was tabulated in the cost analysis.

#### 3.1.5. After-Hours Emergency Calls

In the unconditional model, after-hours emergency calls decreased over time for both study groups at the 12-week (*b* = −0.70, *SE* = 0.13, *p*  < 0.001) and 24-week (*b* = −0.76, *SE* = 0.13, *p*  < 0.001) visits. In the final multivariate model, after-hours emergency calls significantly decreased by the 12-week (*b* = −0.46, SE = 0.21, *p*=0.027) and the 24-week (*b* = −0.54, SE = 0.21, *p*=0.010) time points. Regression coefficients indicated that there was not a significant treatment group difference in after-hours emergency calls at baseline (*p*=0.056). There were also not significant treatment group differences in the change in after-hours emergency calls from baseline to the 12-week (*p*=0.144) or the 24-week time points (*p*=0.178) (Table 5).

### 3.2. Cost Analysis

During the 18 months prior to the use of rtCGM, there were 65 total events by the 33 study participants. For the purposes of comparison, all costs (baseline and at completion of the study) were annualized. We observed a decrease for all events, leading to the substantial projected cost-saving to the payor (private or public health plans).

There were 12 ER visits at baseline; 5 during the 24-week trial. Costs for ER visits from Simeone JC and colleagues provided a range of $972–1,499 per diabetes-related ER visit for persons with T1D [28]. Using a conservative approach, $972 was used in our calculations. Annualized ER visit costs at baseline were $7,776, and annualized costs from the events observed in the study were $9,720.

Hospital admissions for the hyperglycemia management and DKA decreased from 25 events to only one. Hospital admissions for the hyperglycemia management and DKA admissions were estimated at $26,566 per admission [29]. Annualized hospitalization costs at baseline were $442,767, and annualized costs observed in the study were $53,132.

The local EMS cost estimate was ∼$1,300 [30]. The use of EMS was one for the cohort at study baseline and none of these events were observed throughout the study duration. Annualized EMS costs at baseline were $867, and annualized costs observed in the study were zero.

Lastly, the estimated cost per hour for after-hour emergency calls by a board-certified pediatric endocrinologist was calculated at ∼$108 per hour [31]. We conservatively estimated that each call utilized 15 minutes of physician time or $27. After-hour emergency calls decreased from 27 at baseline to four after 24 weeks. The annualized baseline cost was $486 with the annualized cost from the study analysis at $216.

The cost savings estimates are exploratory as rtCGM costs will vary depending on insurance coverage. However, they also may decrease the costs of other blood glucose testing supplies (e.g., test strips). The average list price for one year of Dexcom G6 devices (sensors, transmitters, and a receiver) is estimated at ∼$5,845, with a typical Medicare cost of ∼$2,867 per year [32]. Cost-savings were calculated using the Medicare cost and list price to provide an estimated range for rtCGM cost-saving to any payor. Annualized costs for all baseline diabetes-related events data were $451,895, and after the study, $63,068. Estimated use of rtCGM in this cohort results in a projected annualized cost-savings of $195,943 to $294,864 or 43–65% per year based on Medicare or list pricing for rtCGM, respectively. An estimated annual cost-savings per patient per year would be $5,937 to $8,935.

### 3.3. A1c

**Figure 2:**
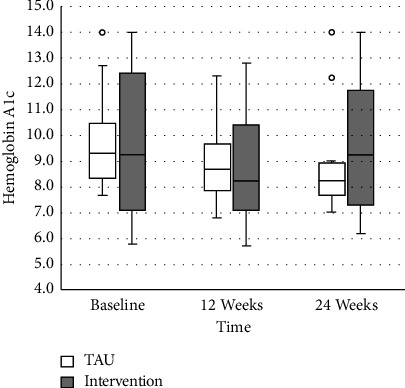
Hemoglobin A1c values vs. time. TAU = treatment as usual.

In the unconditional model, results demonstrated that A1c decreased by 12-week follow-up across all study participants (*b* = −0.91, *SE* = 0.24, *p*  < 0.001). There was a trend of decreased A1c at 24 weeks; however, it was not statistically significant. A1c had not significantly decreased in the total sample at the 24-week follow-up (*p* = 0.191). In the final multivariate model, A1c significantly decreased by the 12-week (*b* = −0.85, *SE* = 0.37, *p* = 0.025) and 24-week (*b* = −0.95, *SE* = 0.37, *p* = 0.013) follow-up time points. Regression coefficients indicated that there was no significant treatment group difference in A1c on average (*p* = 0.842). There was also not a significant treatment group difference in the change in A1c from baseline to the 12-week time point (*b* = −0.10, *SE* = 0.48, *p* = 0.834). There was a significant time intervention interaction effect at the 24-week time point (*b* = 1.03, *SE* = 0.48, *p* = 0.035), meaning that change over time in A1c was significantly different based on the treatment group. Specifically, while there was a decrease in A1c for the TAU group, there was an increase in A1c in the intervention group between baseline and 24 weeks (see Figure 2).

## 4. Discussion

We first hypothesized that rtCGM use would demonstrate decreased diabetes-related events, leading to lower overall health care costs among a unique, high-risk AYA cohort. This aim was supported in our analysis and has direct, practical implications for health care expenditures, especially in marginalized populations where healthcare disparities exist. All diabetes-related events, but especially DKA admissions, are costly to the healthcare system. Targeting high-risk groups has merit that may not transfer to the general T1D population but does demonstrate substantial savings in high-risk AYA with T1D.

Secondly, we anticipated A1c to improve overall and treatment group differences to emerge for those in the intervention group compared to the TAU group. This hypothesis was not supported. We found that A1c improved at 12 weeks but that there was no significant difference at 24 weeks. Of note, A1c demonstrated a slight increase at the 24-week time point for the intervention group compared to the TAU group, which contributed to the overall impact on A1c.

The intervention group had initial improvements to A1c, during which time the intervention phone calls occurred. This effect was not sustained after the intervention period had concluded, and it even worsened. Of note, only 65% of participants completed all three intervention phone calls, and 10% did not complete any (no answer or return call); however, all participants randomized to the intervention group were included in this summary analysis. Baseline A1c and other glucometrics throughout the entire study were above target for most participants (Tables 2–5), which was not unexpected with this high-risk group of 33 AYA. The T1D Exchange Registry has shown an increased risk of DKA in those with higher A1c, independent of CGM, and particularly for those with an A1c ≥9% [3]. A1c rates ≥9% decreased throughout the study, but many sustained elevated A1c and related high-risk for healthcare events; however, decreased events were still observed with the use of rtCGM.

The intervention group results merit further examination to better understand the rationale for worsening glycemic trends at 24 weeks. There are many possible explanations for these results, such as challenges sustaining action/goals without CDCES® reaching out, no further perceived accountability, etc. Our CDCES® study personnel identified several social determinants of health that warrant further investigation in relation to rtCGM outcomes in high-risk AYA, including housing instability, food insecurity, lack of family social support, economic instability, transportation issues, maintaining employment, and limited access to technology, such as an internet connection. Additionally, the impact of diabetes family communication, household chaos, and family conflict should be explored in high-risk AYA and families as these factors have been shown to affect diabetes management, glycemic variability, and optimal CGM use [33, 34]. These findings add to previous research reporting variable outcomes among mobile or phone-based interventions to improve self-management [35–37]. Findings appear to vary based on factors such as patient baseline disease management [37] and a country's economic development [35].

Interestingly, despite a lack of significant change between TAU and intervention groups in any CGM metrics (Tables 3 and 4), when examined collectively, diabetes-related health care events significantly decreased after rtCGM initiation, showing financial value to payors despite lacking a short-term A1c reduction. Specifically, ER visits, hospitalizations, EMS, and after-hours emergency calls decreased across all time points, leading to a conservative estimated cost savings of 43–65% per year in this cohort of AYA with T1D, irrespective of our further intervention. This is consistent with the hypothesis that, at a minimum, access to rtCGM use alone in AYA can drive down health care events. We believe this analysis is a conservative estimate of the cost savings regarding the financial implications of rtCGM use. These findings are consistent with previous research reporting that CGM alone significantly improves diabetes-related clinical outcomes and overall health care costs [1, 7, 17, 22, 23].

## 5. Limitations and Future Directions

While the present study adds further clinical and financial findings to rtCGM use among a high-risk cohort of AYA, it is not without limitations. This protocol was created before the pandemic and stay-at-home orders were in place. The abrupt adoption of telehealth, the accelerated use of technology, remote CGM monitoring, and changes from the normal daily routine likely influenced our recruitment and retention. It is possible the pandemic modified participants' health-seeking behaviors, reducing the frequency of diabetes-related health events. For example, participants may have avoided going to the emergency room due to the perceived heightened COVID-19 risk in healthcare settings. Varied results have been reported during the pandemic regarding glycemic changes and hospitalizations in those with preexisting T1D [38–40]. Additionally, the psychological and socioeconomic sequelae associated with the pandemic may have differentially impacted participants' and families' diabetes management behaviors, family interactions, and responses to rtCGM use.

Another obstacle occurred when statewide changes to rtCGM insurance coverage policies were modified. At the time of project development, our state-funded insurance program did not cover any CGM devices. State-funded CGM coverage was modified shortly before recruitment began, precluding those who had previously used rtCGM from qualifying for our study.

In addition, our small sample size restricted our ability to analyze group differences and additional predictors that may have impacted our intervention outcomes. Inconsistent participation in the pattern management intervention may have impacted the efficacy of this additional tailored support. Additionally, rtCGM is liable for technological difficulties, which may have differentially impacted certain participants' ability to consistently use their devices. For example, the personal cell phone device or internet access of participants and their families may have impacted efficient and accurate rtCGM use and participation in pattern management intervention (e.g., some participants were unable to upload data due to internet access and/or technology challenges). This also likely overlapped with social disparities (e.g., lower income). Finite resources (funding of rtCGM devices) restricted the data collection phase to 24 weeks, limiting our ability to draw longer-term clinical benefits and cost savings.

Lastly, the cost savings are broad estimates and will vary based on unique negotiated rates and rebates (e.g., decreased fee for services at a local ER or hospital; some insurance plans may not reimburse for MD time for after-hour calls; rtCGM rates will vary by the payor, etc.). Costs were primarily driven by reduced hospitalizations, which will vary based on the severity of DKA and any concurrent medical issues. Annualized events for costs could also impact the monetary values; however, the collective estimated cost-saving can still be recognized.

## 6. Conclusions

The economic burden of diabetes, particularly diabetes-related events, is costly for patients, payors, and society. Our findings suggest that rtCGM use may be an effective tool for reducing diabetes-related events, specifically in the most vulnerable, costly cohorts that do not get access to rtCGM technology. When it comes to improving glycemic metrics for high-risk AYA, rtCGM is one piece of the puzzle. Findings related to glycemic health were less clear in this analysis, and additional research is warranted to elucidate the multitude of factors that influence A1c in high-risk AYA with T1D. Future research should examine rtCGM use and social determinants of health, coupled with additional psychological, behavioral, and family systems factors. High-risk AYA likely need more specific, tailored interventions and support to optimize rtCGM use and improve not only costs but glycemic health. Regardless, the present findings demonstrated improvements in diabetes-related events, which are desirable health outcomes, along with cost savings, underscoring the importance of access to rtCGM for this population.

## Figures and Tables

**Table 1:** Participant demographics and clinical characteristics.

Characteristic	Overall	TAU	Intervention
	*N* = 33^1^	*N* = 13^1^	*N* = 20^1^
*Gender*			
Male	17 (51.5%)	6 (46.2%)	11 (55.0%)
Female	16 (48.5%)	7 (53.8%)	9 (45.0%)

*Race*			
White	24 (72.7%)	9 (69.2%)	15 (75.0%)
Black/African American	6 (18.2%)	3 (23.1%)	3 (15.0%)
Native American/American Indian	3 (9.1%)	1 (7.7%)	2 (10.0%)

*Ethnicity*			
Hispanic or latino	9 (27.3%)	4 (30.8%)	5 (25.0%)
Nonhispanic or latino	24 (72.7%)	9 (69.2%)	15 (75.0%)

*Insurance status at baseline*			
Medicaid	21 (63.6%)	10 (76.9%)	11 (55%)
Private	11 (33.3%)	2 (15.4%)	9 (45%)
None	1 (3.0%)	1 (7.7%)	0 (0.0%)

*Insulin management*			
Multiple daily injections	27 (81.8%)	10 (76.9%)	17 (85.0%)
Continuous subcutaneous insulin infusion	6 (18.2%)	3 (23.1%)	3 (15.0%)

*Total diabetes-related events at baseline*			
1	21 (63.6%)	7 (53.8%)	14 (70.0%)
2	5 (15.2%)	3 (23.1%)	2 (10.0%)
3	3 (9.1%)	2 (15.4%)	1 (5.0%)
>3	4 (12.1%)	1 (7.7%)	3 (15.0%)

^1^ *n* (%). TAU = treatment as usual.

**Table 2:** Descriptive statistics of study variables.

Characteristic	Overall, *N* = 33^1^	TAU, *N* = 13^1^	Intervention, *N* = 20^1^
Age at baseline^2^	17.86 ± 2.44	18.33 ± 2.96	17.56 ± 2.07
Age at diabetes diagnosis^2^	10.51 ± 4.14	11.34 ± 4.57	9.97 ± 3.88
Duration of diabetes	7.45 ± 4.30	7.22 ± 4.45	7.59 ± 4.30
A1c at baseline	9.66 ± 2.84	9.75 ± 1.84	9.60 ± 2.58
A1c at 12 W	8.74 ± 1.86	8.90 ± 1.56	8.64 ± 2.06
A1c at 24 W	9.34 ± 2.43	8.81 ± 2.02	9.68 ± 2.66
ER visits at baseline	0.36 ± 0.70	0.54 ± 0.66	0.25 ± 0.72
ER visits at 12 W	0.03 ± 0.17	0.00 ± 0.00	0.05 ± 0.22
ER visits at 24 W	0.12 ± 0.33	0.23 ± 0.44	0.05 ± 0.22
Hospitalizations at baseline	0.76 ± 1.3	0.77 ± 1.42	0.75 ± 1.25
Hospitalizations at 12 W	0.00 ± 0.00	0.00 ± 0.00	0.00 ± 0.00
Hospitalizations at 24 W	0.03 ± 0.17	0.08 ± 0.27	0.00 ± 0.00)
EMS at baseline	0.03 ± 0.17	0.08 ± 0.28	0.00 ± 0.00
EMS at 12 W	0.00 ± 0.00	0.00 ± 0.00	0.00 ± 0.00
EMS at 24 W	0.00 ± 0.00	0.00 ± 0.00	0.00 ± 0.00
After hr emergency calls at baseline	0.76 ± 0.87	0.54 ± 0.66	0.90 ± 0.97
After hr emergency calls at 12 W	0.06 ± 0.24	0.08 ± 0.28	0.05 ± 0.22
After hr emergency calls at 24 W	0.00 ± 0.00	0.00 ± 0.00	0.00 ± 0.00

^1^Mean ± standard deviation. ^2^Variable is in years. *Note.* TAU = treatment as usual. A1c = hemoglobin A1c. ER = emergency room. EMS = emergency medical services. Hr = Hour. 12 W = 12-week study visit. 24 W = 24-week study visit.

**Table 3:** Glycemic outcomes at 12 weeks.

	TAU	Intervention	3*t*-test	*p* value	Cohen's *d*
	*N* = 13^1^	*N* = 19^1+^			
Time rtCGM active (%)	88.84 ± 26.01	85.63 ± 18.73	0.407	0.687	0.146
Time in range 70–180 mg/dL (%)	30.28 ± 16.60	34.20 ± 18.98	−0.603	0.551	−0.217
Mean glucose (mg/dL)	235.92 ± 52.74	224 ± 53.59	0.622	0.539	0.224
Coefficient of variation	32.44 ± 9.49	36.43 ± 11.12	−1.057	0.299	−0.38

*Hypoglycemia*					
Time with glucose 54–69 mg/dL (%)	0.57 ± 1.18	1.17 ± 1.16	−1.441	0.160	−0.519
Time with glucose <54 mg/dL (%)	1.04 ± 3.15	0.41 ± 0.61	0.859	0.397	0.309

*Hyperglycemia*					
Time with glucose 181–250 mg/dL (%)	29.55 ± 10.97	25.79 ± 8.31	1.102	0.279	0.397
Time with glucose >250 mg/dL (%)	38.61 ± 23.57	35.87 ± 24.87	0.312	0.757	0.112

^1^Mean ± standard deviation. ^+^One participant was excluded from this data due to missing rtCGM data at the 24-week study visit. rtCGM = real-time continuous glucose monitoring. *Note.* Data collection period includes the 2 weeks prior to the 12-week study visit.

**Table 4:** Glycemic outcomes at 24 weeks.

	TAU	Intervention	*t*-test	*p* value	Cohen's *d*
	*N* = 13^1^	*N* = 19^1+^			
Time rtCGM active (%)	95.08 ± 5.16	89.72 ± 14.49	1.482^*∗*^	0.151	0.459
Time in range 70–180 mg/dL (%)	33.42 ± 19.73	25.30 ± 20.63	1.241	0.224	0.447
Mean glucose (mg/dL)	231.00 ± 56.05	263.21 ± 71.03	−1.367	0.182	−0.492
Coefficient of variation	33.21 ± 9.55	30.77 ± 10.37	0.674	0.505	0.243

*Hypoglycemia*					
Time with glucose 54–69 mg/dL (%)	0.65 ± 0.96	0.56 ± 0.74	0.294	0.771	0.106
Time with glucose <54 mg/dL (%)	1.05 ± 2.96	0.07 ± 0.21	1.192^*∗*^	0.256	0.522

*Hyperglycemia*					
Time with glucose 181–250 mg/dL (%)	29.95 ± 17.00	19.89 ± 12.09	1.989	0.056	0.709
Time with glucose >250 mg/dL (%)	39.41 ± 24.72	53.14 ± 29.13	−1.390	0.175	−0.500

^1^Mean ± standard deviation. ^+^One participant was excluded from this data due to missing rtCGM data at the 24-week study visit. rtCGM = real-time continuous glucose monitoring. *Note.* Data collection period includes the 2 weeks prior to the 24 week study visit. ^*∗*^Equal variances not assumed.

**Table 5:** A1c mixed effects regression models.

Parameter	A1c		Total events		ER events		Hospitalizations		Emergency calls	
	Coefficient	SE	Coefficient	SE	Coefficient	SE	Coefficient	SE	Coefficient	SE
*Fixed effects*										
(Intercept)	9.75^*∗∗∗*^	0.62	1.92^*∗∗∗*^	0.29	0.54^*∗∗∗*^	0.13	0.77^*∗∗∗*^	0.21	0.54^*∗∗∗*^	0.15
Time (12 W)	−0.85^*∗*^	0.37	−1.85^*∗∗∗*^	0.39	−0.54^*∗∗*^	0.18	−0.77^*∗∗*^	0.29	−0.46^*∗*^	0.21
Time (24 W)	−0.95^*∗*^	0.37	−1.62^*∗∗*^	0.39	−0.31	0.18	−0.69^*∗*^	0.29	−0.54^*∗∗*^	0.21
Intervention	−0.16	0.79	−0.02	0.38	−0.29	0.16	−0.02	0.27	0.36	0.19
Time (12 W) *X* intervention	−0.10	0.48	0.15	0.51	0.34	0.23	−0.02	0.37	−0.39	0.26
Time (24 W) *X* intervention	1.03^*∗*^	0.48	−0.23	0.51	0.11	0.23	−0.06	0.37	−0.36	0.27

*Random effects*										
SD (intercept: participant)	2.01		0.32		0.00		0.21		3.75*e* − 09	
SD (residual)	0.96		1.00		0.45		0.74		0.52	

A1c = hemoglobin A1c. SE = standard error. SD = standard deviation. ER = emergency room. 12 W = 12-week study visit. 24 W = 24-week study visit. ^*∗*^*p*  < 0.05. ^*∗∗*^*p*  < 0.01. ^*∗∗∗*^*p*  < 0.001.

## Data Availability

Additional data questions may be directed to the corresponding author.
